# Potential biotechnological capabilities of cultivable mycobiota from carwash effluents

**DOI:** 10.1002/mbo3.498

**Published:** 2017-07-17

**Authors:** Timothy Sibanda, Ramganesh Selvarajan, Memory Tekere, Hlengilizwe Nyoni, Stephen Meddows‐Taylor

**Affiliations:** ^1^ Department of Environmental Sciences College of Agriculture and Environmental Sciences Florida South Africa; ^2^ Department of Nanotechnology and Water Sustainability College of Science, Engineering and Technology Florida South Africa; ^3^ College of Agriculture and Environmental Sciences Laboratories Florida South Africa

**Keywords:** bioactive, bioresource, extreme environment, fungi, secondary metabolites

## Abstract

Urban life has created man‐made extreme environments like carwashes. These environments have, however, not been sufficiently explored for mycobiota that can be sources of biotechnologically useful products, as has been the case with natural extreme environments. Using a combination of culture and molecular techniques, fungi from carwash effluents was characterized for production of lipase and cellulase enzymes, nonpolar and polar biotechnologically relevant secondary metabolites and hydrocarbon utilization. The isolated fungal strains belonged to the genera *Alternaria*,* Cladosporium*,* Penicillium*,* Peyronellaea*,* Rhizopus*,* Spegazzinia*,* Trichoderma*,* Ulocladium* and *Yarrowia*. Sixty‐six percent (66%) of the fungal isolates were found to be able to metabolize naphthalene and benzanthracene, showing potential for application in bioremediation of hydrocarbon polluted sites. Lipase production by the isolates *Penicillium* sp. BPS3 (2.61 U/ml), *Trichoderma* sp. BPS9 (2.01 U/ml), *Rhizopus* sp. CAL1 (2.05 U/ml), *Penicillium* sp. PCW1 (2.99 U/ml) and *Penicillium* sp. SAS1 (2.16 U/ml) compared well with previously recorded lipase production levels by other fungi. The highest producers of cellulase were *Penicillium* sp. SAS1 (12.10 U/ml), *Peyronella* sp. CAW5 (4.49 U/ml) and *Cladosporium* sp. SAS3 (4.07 U/ml), although these activities were lower than previously reported levels. GC‐MS analysis of the fungal secondary metabolites resulted in identification of 572 compounds, including azulene, methanamine, N‐pentylidene, metoclopramide, and mepivacaine while compounds determined by UHPLC‐MS included 10‐undecen‐1‐ol, piquerol A, 10‐undecyn‐1‐ol, cyclo(leucylprolyl) and rac‐etomidate. These compounds were previously determined to have various activities including anticancer, antibacterial, antifungal, antihypertensive, antidiabetic and anti‐inflammatory properties. The study demonstrated that fungi from carwash effluents are natural sources of some biotechnologically important products.

## 1 INTRODUCTION

Until recently, the search for microbial life in extreme and hyper‐polluted environments has been directed mostly at prokaryotes (Selbmann et al., 2013). However, research is increasingly showing that eukaryotes, and notably fungi, can do particularly well even in stressing environments owing to their ability in colonizing novel environments, unusual resources and forming novel associations that enhance their chances of survival (Gostinčar, Grube, De Hoog, Zalar, & Gunde‐Cimerman, 2010). What makes the study of fungi from extreme and polluted environments particularly interesting is that in a bid to survive in their restrictive environments, they begin to produce compounds with specific survival‐aiding roles especially aimed at stabilizing their membranes and maintaining turgor pressure (Chávez, Fierro, García‐Rico, & Vaca, 2015). Resultantly, these fungi end up synthesizing many molecules which are specific to them, and cannot be produced by fungi in normal, friendly environments. In trying to answer questions surrounding the emergence of antibiotic resistance and the need for new antimicrobials, Büttel et al. (2015) posed the question, “*Can we, by unlocking the hidden potential of fungi, attain new means to gain advantage of this battle*?” As much as this question could have been focused on antibiotics, fungi are a source of many other biotechnologically important molecules ranging from enzymes (Birhanli & Yeşilada, 2013; Elisashvili et al., 2008) to medicinal and therapeutic molecules (Gupta, Saxena, & Goyal, 2014; Hong et al., 2015). Whole cell or culture forms of fungi have also been applied to the bioremediation of metal‐polluted environments (Iram, Zaman, Iqbal, & Shabbir, 2013; Kumar, Singh, Dhir, Sharma, & Mehta, 2014; Siddiquee, Aishah, Azad, Shafawati, & Naher, 2013), biotreatment of raw wastewater (Coulibaly, Gourene, & Agathos, 2004; Novotny et al., 2011), and the bioremediation of persistent pollutants like plastics (Manzur & Limo, 2010), organochlorides (Tekere, Ncube, Read, & Zvauya, 2002) and petroleum hydrocarbons (Makut, Ogbonna, Ogbonna, & Owuna, 2014; Zafra, Absalón, & Cortés‐Espinosa, 2015). While fungi have been shown to be able to colonize most extreme habitats that were previously thought to be inhabited only by prokaryotes, most fungi (with the exception of yeasts) are obligate aerobes and, therefore, would not grow in anaerobic conditions or in environments with temperatures exceeding 70°C (Aguilera, 2013; Cantrell, Dianese, Fell, Gunde‐Cimerman, & Zalar, 2011).

From a microbiological perspective, urban life has created man‐made environments which provide extreme conditions (Gümral et al., 2016). Dishwashers and carwashes have been categorized as some of the man‐made extreme environments housing extremophilic fungi (Zalar, Novak, De Hoog, & Gunde‐Cimerman, 2011). Further, the discharge of pollutants into the environment contributes to environmental toxicity (Spina, Anastasi, Prigione, Tigini, & Varese, 2012), and induces physiological and morphological changes in indigenous microbial flora (Tahir, 2012). This exerts a selective pressure that favors the growth and proliferation of those microorganisms that are able to tolerate and use introduced pollutants to their advantage. Carwash facilities produce and discharge effluents that are characterized by high levels of polycyclic aromatic hydrocarbons (PAHs), heavy metals, high biochemical oxygen demand (BOD) and chemical oxygen demand (COD), and detergents. These effluents have demonstrated toxicity against bacteria (*Vibrio fischeri*), daphnia (*D. magna*), algae (*Selenastrum capricornutum*) and fish (*Poecilia reticulata*) (Tekere, Sibanda, & Maphangwa, 2016). Onsite carwash effluent holding tanks therefore constitute stressed environments which, analogous to natural extreme environments, could be sources of novel mycobiota or mycobiota with potential for production of novel, biotechnologically relevant molecules. To date, no study has yet profiled the mycobiota in this kind of environment and their potential application in biotechnology remains untapped. The aim of this study, therefore, was to characterize fungi from carwash effluents, and to profile and determine the bioresource potential of their secondary metabolites.

## 2 MATERIALS AND METHODS

### 2.1 Sample collection, isolation and characterization of fungi

One litre grab carwash effluent samples were obtained from onsite treatment facilities of five carwash stations in the Johannesburg Metropolitan Municipality in Gauteng Province, South Africa. The sampling stations were code‐named BPS, CAC, CAL, PCW and SAS. The samples were immediately chilled by placing them in cooler boxes containing ice and transported to the laboratory at UNISA Science Campus (Johannesburg) for analysis within 6 hr of collection. Before plating, the samples were shaken to ensure homogeneity after which 100 μl aliquots of each sample were spread plated in triplicate onto freshly prepared potato dextrose agar (PDA) (Sigma Aldrich, Pretoria RSA). The plates were incubated at 27°C for 72 hr. The resultant fungal cultures were purified by subculturing onto fresh PDA plates until axenic cultures were obtained. DNA was then isolated from each culture using a Quick *g*‐DNA extraction kit (Zymo Research, USA) followed by PCR amplification, using ITS 1 and 4 primers described in Bai, Cui, Jie, and Cai (2015). The PCR amplicons (~650 bp) were confirmed by gel electrophoresis and then sent to Inqaba Biotech (Pretoria, RSA) for sequence analysis. The resultant sequences were manually edited with Chromas lite software v2.6.1 (Technelysium Pty Ltd., South Brisbane, Australia) and subjected to BLAST analysis, using the NCBI server to compare the identity of the isolates. The sequences were then used for phylogenetic analysis using the Molecular Evolutionary Genetic Analysis v6.0 (MEGA6) software, using an alignment created with SINA Aligner. A phylogenetic tree was constructed using the maximum likelihood analysis with kimura 2‐parameter model and 1,000 times of bootstrap replications. The fungal sequences were also submitted to the GenBank to obtain accession numbers which are reported in Table 1.

**Table 1:** Results of sequence analysis of fungal ITS chromatograms and accession numbers

Isolate code	Closest similarity	% similarity	Accession number
BPS1	*Peyronellaea* sp.	99	KY073405
BPS2	*Peyronellaea* sp.	99	KY073406
BPS3	*Penicillium expansum*	100	KY073407
BPS4	*Penicillium* sp.	99	KY073408
BPS5	*Alternaria alternata*	100	KY073409
BPS6	*Yarrowia* sp.	99	KY073410
BPS7	*Ulocladium* sp.	99	KY073411
BPS8	*Penicillium chrysogenum*	100	KY073412
BPS9	*Trichoderma* sp.	99	KY073413
BPS10	*Penicillium crustosum*	100	KY073414
CAL1	*Rhizopus* sp.	99	KY073415
CAW1	*Penicillium expansum*	100	KY073416
CAW2	*Peyronellaea* sp.	99	KY073417
CAW3	*Spegazzinia* sp.	99	KY073418
CAW4	*Penicillium* sp.	99	KY073419
CAW5	*Peyronellaea* sp.	99	KY073420
CAW6	*Penicillium adametzioides*	100	KY073421
PCW1	*Penicillium* sp.	99	KY073422
PCW2	*Trichoderma* sp.	99	KY073423
PCW3	*Trichoderma atroviride*	100	KY073424
SAS1	*Penicillium rubens*	100	KY073425
SAS2	*Peyronellaea* sp.	99	KY073426
SAS3	*Cladosporium* sp.	99	KY073427
SAS4	*Peyronellaea* sp.	99	KY073428

### 2.2 Screening for hydrocarbon degradation

A modified protocol of El Hanafy et al. (2015) was used to screen for fungal utilization of hydrocarbons. Briefly, Bushnell‐Hass agar comprising of K_2_HPO_4_ (1 g/L), MgSO_4_ (0.2 g/L), KH_2_PO_4_ (1 g/L), CaCl_2_ (0.02 g/L), NH_4_NO_3_ (1 g/L), FeCl_2_ (0.05 g/L), and Agar (1.25% (w/v)) was prepared in 1 L of distilled water. The agar was poured into 90 mm diameter petri plates which were then overlaid with 100 μl of a 1:1 (v/v) mixture of 0.2 mg/ml naphthalene solution prepared by dissolving 10 mg of naphthalene in 50 ml of methanol and 1 mg/ml benzanthracene prepared by dissolving 10 mg of benzanthracene (Sigma, Pretoria, RSA) in 10 ml of methanol. The polycyclic aromatic hydrocarbon (PAHs) solution was evenly spread over the agar surface using sterile disposable spreaders and the plates left in the lamina flow for the solvent to evaporate, leaving behind a visible thin white layer of PAHs on the surface of the agar. The plates were then spot inoculated with square fungal plugs measuring approximately 1 cm by 1 cm and incubated at 27°C for 1 week. Fungal plugs which grew beyond their original size (>1 cm in diameter) after incubation were deemed positive for hydrocarbon utilization.

### 2.3 Screening and assessment for cellulase and lipase production

The ability of fungal isolates to produce lipase was determined by growing the fungal isolates in rhodamine‐olive oil‐agar medium following a modified protocol of Kumar et al. (2012). The agar medium contained, in g/L, agar‐agar 20, MgSO_4_ 0.2, CaCl_2_ 0.02, KH_2_PO_4_ 1.0, K_2_HPO4 1.0, NH_4_NO_3_ 1.0, FeCl_3_ 1.0, and yeast extract 5.0. The medium was adjusted to pH 7.0, autoclaved and cooled to about 50°C after which 31.25 ml of olive oil and 10 ml of 1.0 mg/ml rhodamine B solution was added with vigorous stirring. It was then poured into petri plates under aseptic conditions and allowed to solidify. The plates were inoculated by placing fungal culture plugs measuring approximately 1 cm^2^ on the agar surface. The plates were incubated for 1 week at 27°C after which they were viewed under UV irradiation where lipase producing strains were identified by the formation of orange fluorescent halos around fungal colonies due to the hydrolysis of substrate. For cellulase screening, agar medium containing 0.2% (w/v) carboxymethylcellulose sodium salt (CMC), 1% agar (w/v) and minimal salt medium (as described above) was prepared and poured into petri plates. Inoculation was then done as explained above, followed by incubation at 27°C for 1 week. Hydrolysis zones were visualized by flooding the plates with 0.1% Congo Red stain (Glass World, Johannesburg, South Africa) and allowing to stand for 15 min followed by destaining with 1 M NaCl.

### 2.4 Analysis for enzyme activity

Fungal isolates which were positive for enzyme production during screening were grown in fermentation media consisting of minimal salt medium supplemented with a mixture of olive oil and CMC‐Na salt for 14 days in a shaking incubator at 27°C. After incubation, cell debris was separated from the supernatant under high speed centrifugation (10,000*g* for 10 min) at 4°C. Part of the supernatant was used for extracellular enzyme activity determination where cellulase activity was determined following the protocol of Worthington and Worthington (1993) while lipase activity was determined following the protocol previously described by Abd‐elhakeem, Elsayed, and Alkhulaqi (2013). One cellulase unit was defined as the amount of enzyme needed to liberate 1 μmol of glucose from cellulose in one hour at pH 5.0 at 37°C after 2 hr incubation time while one lipase unit (U) was defined as the amount of enzyme that could liberate 1 μmol p‐nitrophenol/min under the described assay conditions. All enzyme assays were carried out in triplicate and the average values were calculated.

### 2.5 Analysis for secondary metabolite profiles

The supernatant fraction for secondary metabolite analysis was subjected to solvent extraction (1 part supernatant:1 part solvent, v:v), using a mixture of chloroform and methanol (1:1, v/v) and shaken for 12 hr at 120 rpm and 25°C. The solvent fraction was then separated from the aqueous fraction after which the solvent fraction was evaporated to dryness *in vacuo* at 80°C and the residue reconstituted in a mixture of 1:1 (v:v) acetonitrile and hexane. The acetonitrile fraction was used to analyze for polar secondary metabolites using ultra‐high performance liquid chromatography mass spectrophotometer (UHPLC‐MS) (Compass otofSeries 1.9, Bruker Instrument: Impact II) while the hexane fraction was used to analyze for nonpolar compounds, using gas chromatography mass spectrometer (GC‐MS) (Pegasus 4D GCxGC‐TOFMS, Leco Corporation).

For GC analysis, an HP‐5MS fused silica capillary column (30 m, 0.25 mm i.d., 0.25 μm film, cross‐linked to 5% phenyl methyl siloxane stationary phase) was used. The GC conditions were as follows: ionization energy was set at 70 eV, helium (He) was used as the carrier gas, flow rate was set at 1 ml/min, injection volume at 2 μl, injection temperature at 250°C, and ion source temperature at 200°C. The oven temperature was initially set at 50°C for 5 min, then increased to 260°C (ramp: 4°C/min) and held for 5 min. For MS, ionization energy was set at 70 eV, scan interval at 0.5 s, fragments at 45 to 450 kD and solvent delay at 0 to 2 min. The identification of compounds was based on comparison of their mass spectra with the National Institute of Standards and Technology (NIST 2014) library.

For UHPLC‐MS analysis, the column was Acquity UPLC BEH C18 1.7 μm, diameter 2.1 × 100 mm (Miscrosep Waters, Johannesburg, RSA) while the solvents were 0.1% formic acid (FA) in water and 0.1% FA in acetonitrile. The column flow was set at 0.3 ml/min, column oven temp at 35°C, draw speed at 2 μl/s with a total injection volume of 2 μl. Mass Spectrometer (MS) conditions were set at a mass range of 50–1,600 m/z, capillary 4,500 v, dry gas 8 L/m, dry temp 220°C, ion energy 4.0 eV, collision energy 7.0 eV, cycle time 0.5 s. Data analysis was done, using the Bruker Software (Bruker Compass Data Analysis 4.3, Bruker Daltonik GmbH 2014).

## 3 RESULTS

**Figure 1:**
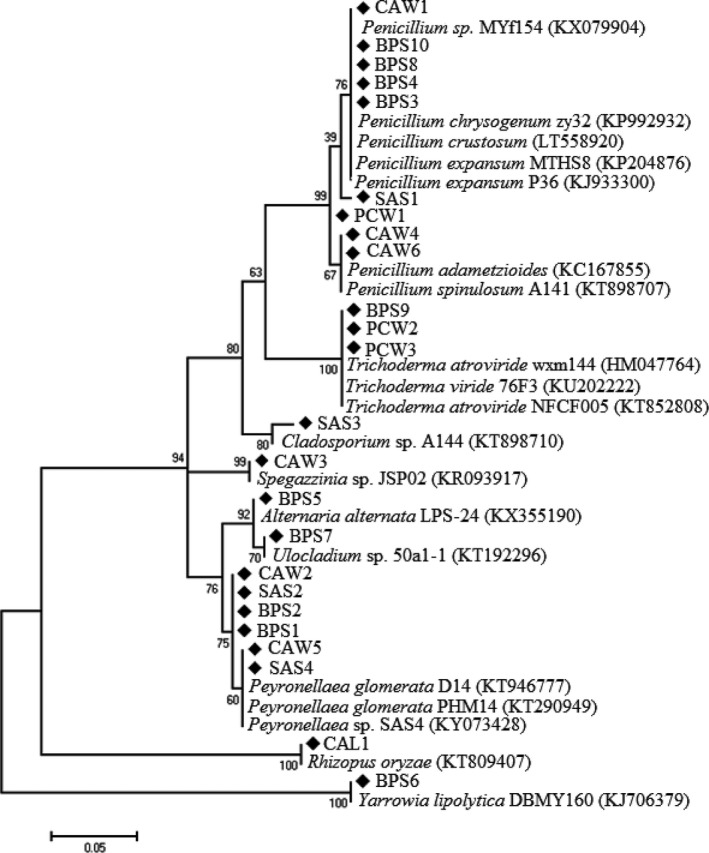
Maximum likelihood distance‐based phylogenetic tree of ITS gene sequences. Bootstrap values are shown on appropriate branches. Fungal isolates from this study are code named while compared fungal strains are written in full (accession numbers in parenthesis)

The physicochemical parameters of the carwash effluents were determined and separately reported in (Tekere et al., 2016). A total of 24 fungal isolates were isolated. Sequencing and phylogenetic analysis of the ITS sequences showed that the 24 isolates belonged to nine different genera namely *Alternaria*,* Cladosporium*,* Penicillium, Peyronellaea, Rhizopus, Spegazzinia, Trichoderma, Ulocladium,* and *Yarrowia*. Of these genera, *Penicillium* contributed 37.5% of the total isolates. *Penicillium* also dominated in terms of coverage since it was found in all car wash samples except CAL, from which only one type of fungal isolate was obtained, *Rhizopus*. Of the total fungal isolates obtained, 45.5% were present in BPS samples, which was the largest number of fungal variants isolated from a single carwash sample. The most uncommon genera were *Rhizopus*,* Ulocladium*,* Spegazzinia*,* Yarrowia*,* Alternaria*, and *Cladosporium* each of which made up only 4.17% of the total isolates. In all instances, the percentage similarity was ≥99%. Details of sequencing and phylogenetic analysis are given in Table 1 and Figure 1 respectively.

Of the 24 fungal isolates, two could neither produce either cellulase or lipase nor could they utilize hydrocarbons as a carbon source. Those that could produce either or both of the enzymes and/or utilize hydrocarbon as a sole carbon source are listed in Table 2. Enzyme activity (U/ml) for each isolate that tested positive during enzyme screening was also determined using suitable substrate and is detailed in Table 2. Sixty six percent (66%) of the isolates were able to utilize hydrocarbon as a carbon source, followed by lipase production at 54.17%. Only two isolates, *Trichoderma* sp. BPS9 and *Penicillium* sp. SAS1 could produce both enzymes (cellulase and lipase) and utilize hydrocarbon as the sole source of carbon while the rest of the isolates could either produce both enzymes but fail to degrade hydrocarbons or produce one of either cellulase or lipase as well as utilize hydrocarbon as a sole source of carbon. The cellulase enzyme activity of *Penicillium* sp. SAS1 was higher than that of the commercial cellulase from *Aspergillus niger* (Sigma Aldrich, South Africa) which had a value of 5.93 U/ml while the cellulase enzyme activity of isolates *Cladosporium* sp. SAS3 (4.49 U/ml) and *Peyronellaea* sp. CAW5 (4.07 U/ml) compared well to that of the positive control. For lipase enzyme activity, *Penicillium* sp. PCW1 had higher activity (2.99 U/ml) than the activity of a commercial lipase from *Rhizopus oryzae* (Sigma Aldrich, South Africa) which had an activity value of 2.94 U/ml. However, lipase production by *Penicillium* sp. BPS3 (2.61 U/ml), *Trichoderma* sp. BPS9 (2.01 U/ml), *Rhizopus* sp. CAL1 (2.05 U/ml) and *Penicillium* sp. SAS1 (2.16 U/ml) still compared well with the production levels of the positive control.

**Table 2:** Hydrocarbon utilization and enzyme production profiles of the fungal isolates

Assays			
Isolate name	Cellulase activity^a^	Lipase activity^a^	Hydrocarbon utilization
*Peyronellaea* sp. BPS1	NA	NA	+
*Peyronellaea* sp. BPS2	0.09 ± 0.01	NA	+
*Penicillium* sp. BPS3	NA	2.61 ± 0.2	+
*Penicillium* sp. BPS4	0.30 ± 0.02	1.89 ± 0.03	‐
*Alternaria* sp. BPS5	NA	1.41 ± 0.01	+
*Yarrowia* sp. BPS6	NA	1.45 ± 0.01	‐
*Ulocladium* sp. BPS7	0.29 ± 0.04	NA	+
*Penicillium* sp. BPS8	NA	1.42 ± 0.01	‐
*Trichoderma* sp. BPS9	0.10 ± 0.01	2.01 ± 0.1	+
*Penicillium* sp. BPS10	NA	1.36 ± 0.05	+
*Rhizopus* sp. CAL1	NA	2.05 ± 0.04	+
*Penicillium* sp. CAW1	0.27 ± 0.03	NA	‐
*Spegazzinia* sp. CAW3	1.46 ± 0.1	NA	+
*Penicillium* sp. CAW4	NA	NA	+
*Peyronellaea* sp. CAW5	4.49 ± 0.2	NA	‐
*Penicillium* sp. PCW1	NA	b ^B^2.99 ± 0.6	+
*Trichoderma* sp. PCW2	NA	1.69 ± 0.07	+
*Trichoderma* sp. PCW3	NA	1.42 ± 0.2	+
*Penicillium* sp. SAS1	b ^A^12.10 ± 0.4	2.16 ± 0.03	+
*Peyronellaea* sp. SAS2	NA	1.71 ± 0.04	+
*Cladosporium* sp. SAS3	4.07 ± 0.1	NA	+
*Peyronellaea* sp. SAS4	0.38 ± 0.01	NA	‐

a Enzyme activity in U/ml.

b Enzyme activity higher than the activity of (A) cellulase positive control [5.93 U/ml] and (B) lipase positive control [2.94 U/ml].

+ shows ability of the fungal strain to utilize hydrocarbon as sole carbon source.

– shows inability of the fungal strain to utilize hydrocarbon as sole carbon source.

Secondary metabolite determination by GC‐MS resulted in 572 compounds after a similarity cutoff of 800 was done. The compounds belonged to different families including aldehydes, alcohols, esters, amines, amides, alkanes and alkenes, aromatic and cyclic hydrocarbons (including the meth‐, eth‐, prop‐, but‐, pent‐, hex‐, hept‐ , and oct‐ hydrocarbons), oxygenous heterocyclic compounds, ketones, and carboxylic acids. Metabolite‐based cluster and principal component analysis (PCA) using SIMCA version 14 (Umetrics, Umeå, Sweden) (plot not shown) showed *Penicillium* sp. BPS8 to produce some unique metabolites, making it different from the rest of the isolates. *Yarrowia* sp. BPS6 also showed the same pattern of metabolite production as *Penicillium* sp. BPS8, though with a much reduced distance from other isolates. *Peyronellaea* sp. SAS2, *Trichoderma* sp. PCW2, *Penicillium* sp. CAW1, *Rhizopus* sp. CAL1, *Peyronellaea* sp. SAS4, *Penicillium* sp. PCW1 and *Penicillium* sp. SAS1 were shown to be metabolically different from the rest of the other isolates. *Penicillium* sp. SAS1 and *Penicillium* sp. PCW1 as well as *Didymella* sp. SAS2 and *Trichoderma* sp. PCW2 were, however, metabolically closer to each other. Further, PCA showed that *Penicillium* sp. BPS3, *Penicillium* sp. BPS10 and *Peyronellaea* sp. CAW5 were almost metabolically similar, judging from their metabolite profiles.

**Figure 2:**
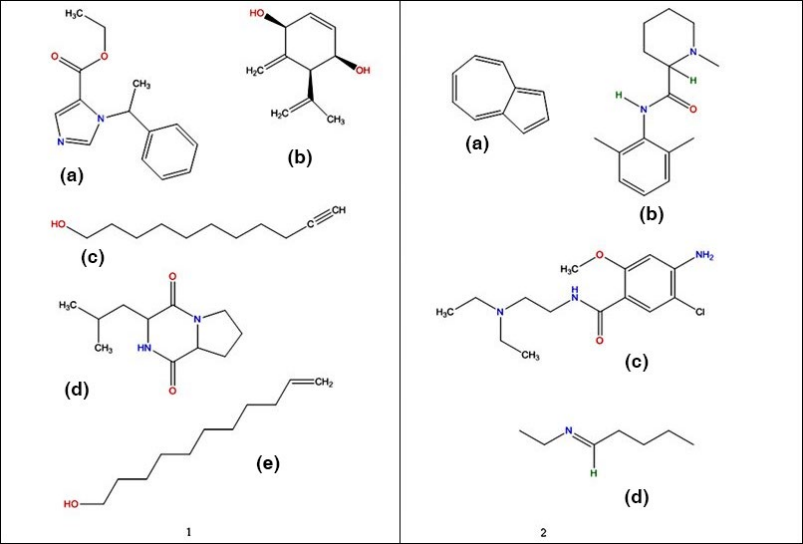
Molecular structures of fungal secondary metabolites determined by (1) UHPLC‐MS [a] rac‐etomidate, [b] piquerol A, [c] 10‐undecyn‐1‐ol, [d] cyclo(leucylprolyl) and [e] 10‐undecen‐1‐ol and (2) GC‐MS [a] azulene, [b] mepivacaine, [c] metoclopramide and [d] methanamine, N‐pentylidene

Some of the secondary metabolites identified by GC‐MS are given in Table 3 while Figure 2 shows the structures of selected compounds identified by GC‐MS and UHPLC‐MS. Selection of compounds for presentation in results and discussion was based on principal component analysis where the compounds which influenced dispersion, mostly because they were only produced by a few fungal strains.

**Table 3:** Some of the fungal secondary metabolites identified by GC‐MS and their potential applications

aCompound	Producing organisms	Known uses/applications
Azetidine	*Yarrowia* sp. BPS6, *Ulocladium* sp. BPS7, *Penicillium* sp. BPS3, *Rhizopus* sp. CAL1 and *Penicillium* sp. PCW1	Azetidines harbor antihypertensive, anti‐inflammatory, antiarrhythmic and antidepressant properties Ojima et al. (1991).
Azulene	*Penicillium* sp. BPS8	Substituted azulenes are widely applied in medicinal chemistry as antiulcer, antidiabetic, anticancer, anti‐erectile dysfunction and antiarrhythmic agents Cowper, Jin, Turton, Kociok‐Köhn, & Lewis (2016); Teufel (1999).
Mepivacaine	*Rhizopus* sp. CAL1	Used as an anaesthetic agent in dentistry AAPD (2009); Brockmann (2014); Haas (2002)
Methanamine, N‐pentylidene	*Trichoderma* sp. PCW2	Identified as an essential component in the bioactivity of some plant extracts Jananie, Priya, & Vijaya Lakshmi (2012).
Metoclopramide	*Penicillium* sp. CAW1	Metoclopramide is used to treat nausea and vomiting, especially after surgery or chemotherapy. However, use of this drug is now restricted owing to its side effects European Medicines Agency (2013); van der Meer, Venhuizen, Heyland, & van Zanten (2014).
Oxazole	*Penicillium* sp. BPS10	Oxazoles have hypoglycemic, antibacterial and anti‐inflammatory activities in addition to being precursors to the building of natural and pharmaceutical products and synthetic intermediates Rauf & Farshori (2012); Smith & Balskus (2002).
Phenylethyl alcohol	All except *Yarrowia* sp. BPS6 and *Penicillium* sp. SAS1	Besides its use in the manufacture of synthetic rose perfumes and cosmetic formulations, this alcohol is also used an antimicrobial preservative in pharmaceutical products like nasal sprays Reza, Fereshteh, & Saman (2015).
Propiolactone	*Penicillium* sp. CAW1	Beta‐propiolactones are used an inactivating reagent in the production of influenza vaccines (inactivated influenza virus) Bonnafous et al. (2014); Uittenbogaard, Zomer, Hoogerhout, & Metz (2011).
4‐Trifluoroacetoxyhexadecane	*Penicillium* sp. BPS10	4‐Trifluoroacetoxyhexadecane has been classified as a fluro compound which has antimicrobial activities Sarada, Jothibai Margret, & Mohan (2011).
1,3,4‐Oxadiazole‐2(3H)‐thione, 3‐(4‐morpholylmethyl)‐5‐phenoxymethyl‐	*Trichoderma* sp. PCW2	No known uses.

a All compounds were determined following a similarity cutoff of 800 of the GC‐MS output.

Of the secondary metabolites analyzed by UHPLC‐MS, five were selected for further discussion (Figure 2(1)) based on dispersion and distance from other compounds following principal component analysis. Cyclo(leucylprolyl) and rac‐etomidate were produced by all the 20 fungal isolates while 10‐undecyn‐1‐ol was produced by the isolate *Penicillium* sp. SAS1 only. Isolate *Ulocladium* sp. BPS7, *Peyronellaea* sp. CAW5, *Penicillium* sp. SAS1 and *Penicillium* sp. BPS3 all produced 10‐undecen‐1‐ol while piquerol A was produced by isolates *Trichoderma* sp. PCW2, *Peyronellaea* sp. SAS2, *Trichoderma* sp. PCW3 and *Penicillium* sp. BPS3. GC‐MS and UHPLC‐MS detection of the compounds was only qualitative; hence information relating to the quantities of the compounds is not available.

## 4 DISCUSSION

Numerous studies have reported the existence of fungi in pollution‐stressed and extreme environments (Aguilera, 2013; Aristi et al., 2015; Cantrell, Casillas‐Martínez, & Molina, 2006; Cantrell et al., 2011). *Aspergillus*,* Penicillium*,* Curvularia*,* Fusarium*,* Microsporum*,* Trichoderma*,* Rhizoctonia*,* Nigrospora*, and *Chaetophoma* have previously been isolated from raw refinery effluents (Machido, Yakubu, & Ezeonuegbu, 2014). However, to our knowledge, this is the first study to not only report on the existence of fungi in carwash effluents but to also characterize such fungi and profile their secondary metabolites. Since carwash effluents are characterized by high concentrations of heavy metals, polycyclic aromatic hydrocarbons, and detergents (Tekere et al., 2016), it follows that the presence of fungi in such environments underlines their potential as agents of bioremediation of environments polluted by any of these pollutants. The excellent potential of fungi to immobilize toxic metals by a number of means among them the formation of insoluble metal oxalates, biosorption and/or chelation onto melanin‐like polymers has previously been reported (D'Annibale, Rosetto, Leonardi, Federici, & Petruccioli, 2006).

Further, the demonstrated ability of most of the fungal isolates in this study, including *Peyronellaea* sp. BPS1, *Peyronellaea* sp. BPS2, *Penicillium* sp. BPS3, *Alternaria* sp. BPS5, *Ulocladium* sp. BPS7, *Trichoderma* sp. BPS9, *Penicillium* sp. BPS10, *Rhizopus* sp. CAL1, *Spegazzinia* sp. CAW3, *Penicillium* sp. CAW4, *Penicillium* sp. PCW1, *Trichoderma* sp. PCW2, *Trichoderma* sp. PCW3, *Cladosporium* sp. SAS3, *Peyronellaea* sp. SAS2, and *Penicillium* sp. SAS1 to utilize hydrocarbon as a sole source of carbon buttresses this argument. While hydrocarbon utilization screens were only qualitative, quantitative cellulase and lipase production assays showed *Penicillium* sp. SAS1 and *Penicillium* sp. PCW1 to be the highest producers of cellulase and lipase, respectively. Lipase production by five of our isolates, *Penicillium* sp. BPS3 (2.61 U/ml), *Trichoderma* sp. BPS9 (2.01 U/ml), *Rhizopus* sp. CAL1 (2.05 U/ml), *Penicillium* sp. PCW1 (2.99 U/ml) and *Penicillium* sp. SAS1 (2.16 U/ml) compared well with the findings of Silva, Mutidieri, Schrank, and Vainstaein (2005) who reported lipase activity ranging from 1.64 U/ml to 4.90 U/ml for the fungus *Metarhizium anisopliae* using different substrates. However, our findings could not compare to the hydrolysis activity of filamentous fungi isolates reported by Pacheco et al. (2015) who reported activities ranging from 89 U/ml to 361 U/ml, using various substrates. Lipase production was targeted in this study because lipase based technologies for the synthesis of novel compounds is on the increase (Andualema & Gessesse, 2012). Lipases are arguably one of the enzymes with the broadest spectrum of applications ranging from food industry, oil and fat industry, detergent industry, leather industry, pulp and paper industry, detergent industry, cosmetic industry, biodiesel production as well as in organic chemistry (Andualema & Gessesse, 2012). Because of their broad applications in enzyme technology, research aimed at discovering microbial producers of lipases and other enzymes from previously unexplored environments should be encouraged.

The highest producers of cellulase in our study were *Penicillium* sp. SAS1 (12.10 U/ml), *Peyronellaea* sp. CAW5 (4.49 U/ml) and *Cladosporium* sp. SAS3 (4.07 U/ml). These, however, could only compare well to the low tier cellulase producing fungi isolates reported by Lakshmi Sri and Narasimha (2012) whose cellulase activity ranged from 5.55 to 5.56 U/ml using carboxymethylcellulose as the substrate. Otherwise they also reported CMCase activity of 43.32 to 64 U/ml/h from their more active isolates. It is expected that the nature of environment from which they obtained their isolates (forest litter soil) was more suitable for cellulolytic fungal isolates than carwash effluents, hence the differences between our findings.

Some fungi isolated in this study, for instance *Penicillium*,* Cladosporium* and *Yarrowia* belong to the genera of fungi that have previously been characterized in extreme environments (Gunde‐cimerman & Zalar, 2011). Additionally, the dominance of *Penicillium* both in terms of frequency and ubiquity suggests that it has the metabolic versatility which enables it to colonize stressing environments. Ezzouhri, Castro, Moya, Espinola, and Lairini (2009) also reported the ability of *Penicillium* sp. to grow in media contaminated with various metals including cadmium, which was able to inhibit the growth of other fungal strains. Besides their presumed metabolic plasticity, the production of conidia in huge quantities by a large number of *Penicillium* sp. could also account to the ubiquity of this fungus in many carwashes. This versatility can be exploited for biotechnological ends such as production of enzymes, bioactive pharmaceuticals and other natural biotechnologically important compounds. Natural products have the distinct advantage of a greater structural diversity compared to synthetic chemical compounds, making them potentially infinite resources of chemical diversity and biological activities (Chávez et al., 2015). Already, our results show that of all the four selected secondary metabolites determined by UHPLC‐MS, there was a *Penicillium* species among the producers of each of the compounds.

Among these UHPLC‐MS determined compounds, piquerol A is a traditionally known plant extract (produced by a herbaceous shrub *Piqueria trinervia*) that is commonly used as an antipyretic, antimalarial and antirheumatic medicine (Hennig, Garcia, Giannis, & Bussmann, 2011; Mendoza, Jimenez, & Lotina‐Hennsen, 1994). While previous studies have linked bio‐production of piquerol A and its derivatives only to plants, our results reveal that *Trichoderma* sp. PCW2, *Peyronellaea* sp. SAS2, *Trichoderma* sp. PCW3 and *Penicillium* sp. BPS3 all produced this compound. The compound 10‐undecyn‐1‐ol, which was produced only by *Penicillium* sp. SAS1 has previously been identified by GC‐MS as one of the phytochemical constituents of the plant *Sarcostemma secamone* (Kumari, Muthukumarasamy, & Mohan, 2012). This compound, which was also isolated in the leaf extracts of *Azadirachta indica* has limited solubility in water and has been determined to have antifungal activity (Neoh, Tanimoto, Ikefuji, Yoshii, & Furuta, 2008). Prior to this study, there has not been a report of the production of 10‐Undecyn‐1‐ol by a *Penicillium* species or by microorganisms. Similarly, the compound 10‐undecen‐1‐ol which was produced by the isolates *Ulocladium* sp. BPS7, *Peyronellaea* sp. CAW5, *Penicillium* sp. SAS1 and *Penicillium* sp. BPS3 have previously been identified as a constituent of the plant *Machilus zuihoensis* (Ho, Liao, & Su, 2012). The essential oils of this plant are known to harbor antimicrobial activity. Since the output of bioactive ingredients from herbaceous plants is limited and would need extensive plantations to meet market demand (Zhang, Huang, Ye, Shi, & Zhang, 2015), biotechnological approaches targeting producing microorganisms or their genes can be employed to amplify production of these molecules. The two other compounds, rac‐etomidate and cylco(leucylprolyl) were produced by twenty fungal isolates. Since the 70s, etomidate has been used as an agent for intubation and procedural sedation in clinical practice though recent in vitro studies (Wu et al., 2011) have proven its capacity to induce cytotoxic effects and increase production of apoptosis associated proteins. Literature that links etomidate production to mycobiota could not be found, however. The compound cyclo(leucylprolyl) has previously been identified as a secondary metabolite of *Enterobacter cloacae* harboring weak antimicrobial activity (Mohammed et al., 2013). However, microbial production of the L isomer of this compound, cyclo(l‐leucyl‐l‐prolyl), has been reported and shown to inhibit aflatoxin production by *Aspergillus parasiticus* (Yan, Song, Sakuno, & Nakajima, 2004) as well as inhibit the growth of several fungal species (Ki‐Hyeong, 2003).

While using online libraries like Chemspider, KEGG and Pubchem to identify the compounds found by UHPLC‐MS, as many as a third of the compounds showed ‘no hits’, a clear indication that microbial secondary metabolites present an infinitely large resource base for potential biotechnologically important molecules which are currently not known. The ‘no hits’ phenomena could also point to the fact that only a fraction of microbial secondary metabolites have been positively identified to date, with most having as yet to be identified and exploited. Besides, most man‐made extreme and moderately extreme environments like car washes have received less or no attention compared to natural extreme environments like the deep ocean sediments, thermal springs, hypersaline solar salterns and arid desserts to mention a few, and the metabolite profiles of organisms from these environments may not match what is already known from other environments. Since microbial production of secondary metabolites is largely influenced by their mechanisms of coping with the presence of certain specific stressors in their environments and are, therefore, unique to those environments, it follows that previously unexplored synthetic extreme and hyper‐polluted environments present untapped sources of novel bioactive molecules.

As was the case with some UHPLC‐MS determined compounds, production of some of the compounds determined by GC‐MS analysis (Table 3) has previously been linked only to plants. Azulene for example is known to be produced either by plants from where it is obtained by distillation or by chemical synthesis (Hezekiah, Labunmi, & Joseph, 2015; Teufel, 1999). Azetidine is another compound which, despite its intriguing biological activities, is known to be contained in the phytosiderophore mugineic acid of some grasses or a marine sponge extract, penaresidin A (Feula et al., 2013). In fact, Ojima et al. (1991) argue that synthesis of the azetidine skeleton is very difficult owing to its ring strain. However, this study has demonstrated fungal production of both azulene by *Penicillium* sp. BPS8 and azetidine by several fungal strains including *Yarrowia* sp. BPS6, *Ulocladium* sp. BPS7, *Penicillium* sp. BPS3, *Rhizopus* sp. CAL1 and *Penicillium* sp. PCW1. While synthetic production of these compounds may offer a cheap and convenient alternative, resorting to natural production of the same brings with it the unparalleled structural diversity richness which offers even more alternatives to produce chiral compounds should there be need to improve on their biological activities. What needs to be noted, however, is that the bioactivities of volatile compounds such as those identified by GC‐MS (Table 3) and to some extent, UHPLC‐MS, may be dependent on the synergistic effects of other compounds produced by other genes in a particular gene cluster. Unlocking of the unknown fungal bioresources therefore requires the use of computational tools to identify conserved features in genes which are involved in secondary metabolite production, which can then be expressed in heterologous host fungal species in order to identify novel products.

To increase the accuracy of compound identification by both UHPLC‐MS and GC‐MS, only compounds whose spectra resulted in a probability score of one (1) when compared to compounds in reference libraries were chosen for presentation and discussion. However, the plethora of secondary metabolites identified in this study may not reflect the complete metabolite fingerprint of the fungi from the studied environments as some thermolabile compounds could have been lost prior to analysis due to the use of high temperatures (80°C) during solvent extraction.

## 5 CONCLUSION

The bioresource potential of mycobiota from carwash effluents was investigated for the first time using a combination of bioassays and GC‐MS and UHPLC‐MS analysis of secondary metabolites. Our results demonstrated potential application of these mycobiota to bioremediation of hydrocarbon contaminated environments, but limited cellulase and lipase activity. Most importantly, the study demonstrated that fungi from carwash effluents are sources of some potential biotechnologically important products. Further, mycobiota from these environments are potential sources of novel compounds which can contribute to biotechnology.

## CONFLICT OF INTEREST

The authors wish to declare no conflict of interests.

## ETHICS AND CONSENT TO PARTICIPATE

Not applicable.

## AVAILABILITY OF DATA AND MATERIALS

The datasets supporting the conclusions of this article are available in the GenBank repository, [https://www.ncbi.nlm.nih.gov/nuccore/KY073405-KY073428].
